# 

**Published:** 1899-10

**Authors:** 


### CONTENTS.

ORIGINAL COMMUNICATIONS—

THE PATHOLOGY OF FEVER. By J. Clarence Johnson, M.D., Atlanta, Ga. .......... 505
CLIMATOLOGY AND SANITATION. By T. S. Hopkins, M.D., Thomasville, Ga. .......... 513
APPENDICITIS. By Hugh F. Lorimer, M.D., Fair Haven, Ohio. .......... 522
A REPORT OF THREE SURGICAL CASES, WITH COMMENTS. By E. C. Davis, M.D., Atlanta, Ga. .......... 530
PENETRATING WOUNDS OF THE EYE. By A. Bethune Patterson, M.D., Atlanta, Ga. .......... 533
REPORT OF TWO CASES OF TYPHOID FEVER. By J. M. McClanahan, M.D., Retreat, S. C. .......... 537
SUSPENSION OF THE UTERUS BY INTRAMURAL SHORTENING OF THE ROUND LIGAMENTS. By Geo. H. Noble, M.D., Atlanta, Ga. .......... 537

SOCIETY REPORTS—

ATLANTA SOCIETY OF MEDICINE .......... 547

EDITORIAL NOTES AND COMMENTS—

TO SUBSCRIBERS .......... 552
THE MEDICAL WITNESS .......... 552
MEDICAL NEWS .......... 554

NEWS AND NOTES .......... 555
CONTENTS CONTINUED .......... x

